# Benzene Exposure Leads to Lipodystrophy and Alters Endocrine Activity *In Vivo* and *In Vitro*

**DOI:** 10.3389/fendo.2022.937281

**Published:** 2022-07-13

**Authors:** Ying Cui, Ziying Mo, Penglei Ji, Jingyi Zhong, Zongxin Li, Daochuan Li, Lina Qin, Qilong Liao, Zhini He, Wei Guo, Liping Chen, Qing Wang, Guanghui Dong, Wen Chen, Yongmei Xiao, Xiumei Xing

**Affiliations:** ^1^ Department of Toxicology, School of Public Health, Sun Yat-sen University, Guangzhou, China; ^2^ Zhongshan School of Medicine, Sun Yat-sen University, Guangzhou, China; ^3^ Department of Occupational and Environmental Health, School of Public Health, Sun Yat-sen University, Guangzhou, China; ^4^ School of Public Health, Food Safety and Health Research Center, Southern Medical University, Guangzhou, China; ^5^ State Key Laboratory of Conservation and Utilization of Bio-Resources in Yunnan and Center for Life Science, School of Life Sciences, Yunnan University, Kunming, China

**Keywords:** benzene, white adipose tissue, lipodystrophy, endocrine function, adipokine

## Abstract

Benzene is a ubiquitous pollutant and mainly accumulates in adipose tissue which has important roles in metabolic diseases. The latest studies reported that benzene exposure was associated with many metabolic disorders, while the effect of benzene exposure on adipose tissue remains unclear. We sought to investigate the effect using *in vivo* and *in vitro* experiments. Male adult C57BL/6J mice were exposed to benzene at 0, 1, 10 and 100 mg/kg body weight by intragastric gavage for 4 weeks. Mature adipocytes from 3T3-L1 cells were exposed to hydroquinone (HQ) at 0, 1, 5 and 25 μM for 24 hours. Besides the routine hematotoxicity, animal experiments also displayed significant body fat content decrease from 1 mg/kg. Interestingly, the circulating non-esterified fatty acid (NEFA) level increased from the lowest dose *(p*_trend_ < 0.05). Subsequent analysis indicated that body fat content decrease may be due to atrophy of white adipose tissue (WAT) upon benzene exposure. The average adipocyte area of WAT decreased significantly even from 1 mg/kg with no significant changes in total number of adipocytes. The percentages of small and large adipocytes in WAT began to significantly increase or decrease from 1 mg/kg (all *p* < 0.05), respectively. Critical genes involved in lipogenesis and lipolysis were dysregulated, which may account for the disruption of lipid homeostasis. The endocrine function of WAT was also disordered, manifested as significant decrease in adipokine levels, especially the leptin. *In vitro* cell experiments displayed similar findings in decreased fat content, dysregulated critical lipid metabolism genes, and disturbed endocrine function of adipocytes after HQ treatment. Pearson correlation analysis showed positive correlations between white blood cell (WBC) count with WAT fat content and plasma leptin level (*r* = 0.330, 0.344, both *p* < 0.05). This study shed light on the novel aspect that benzene exposure could induce lipodystrophy and disturb endocrine function of WAT, and the altered physiology of WAT might in turn affect benzene-induced hematotoxicity and metabolic disorders. The study provided new insight into understanding benzene-induced toxicity and the relationship between benzene and adipose tissue.

## Introduction

Benzene is an essential industrial material, which is widely used in the rubber industry, petrochemical industry, interior decoration and other industries. Benzene is also a widespread environmental pollutant. Benzene could affect the hematopoietic system, cause corresponding clinical symptoms, and even induce aplastic anemia and leukemia (1). As early as 1979, benzene was classified as a class I carcinogen by the International Agency for Research on Cancer (IARC) (2). Thus, during the past decades, researchers have mainly focused on the hematotoxicity and carcinogenicity of benzene. However, recent studies showed a growing concern about the potential impact of benzene on metabolic diseases, such as insulin resistance (3, 4) and cardiovascular diseases (5). Adipose tissue has been recognized as a highly active metabolic and endocrine organ, and its dysfunction is reported to be involved in the development and/or progression of metabolic disorders (6). Either excessive or insufficient adipose tissue is reported to cause insulin resistance and related metabolic diseases (7). In addition, studies indicated that adipose tissue can secrete a variety of adipokines, such as leptin and adiponectin. And the adipokines may play important roles in regulating energy balance, glucose and lipid homeostasis, and even functions of other organs (8, 9). It is well-known that benzene is a high lipophilic compound mainly distributed in lipid-rich tissues and organs such as the liver, bone marrow and adipose tissue. Research of the past decade mostly focused on the adverse effect of benzene on liver and bone marrow, while that of adipose tissue remains poorly understood. Thus, it is necessary to explore the effect of benzene exposure on adipose tissue. The findings may help to understand the role of adipose tissues in benzene-induced adverse effects including metabolic disorders.

The latest findings indicate that the role of adipose tissue is complicated. Adipose tissue can be roughly divided into three types, white fat, brown fat and beige fat. The white fat can be further divided into subcutaneous adipose tissue and visceral adipose tissue according to the anatomical location (10). More and more studies indicate that adipose tissue is not only an organ for energy storage and heat production but also has other important biological functions (11). Adipose tissue is rich in lipids, which makes it prone to be a potential site for the accumulation of lipophilic pollutants, such as benzene and some persistent organic pollutants (POPs) (12). What’s more, many recent studies have shown that adipose tissue is not only an accumulation site, but also a target organ of some lipophilic pollutants, and even participates in the regulation of chemical toxicity (11). It is reported that a variety of lipophilic chemicals, such as bisphenol A (BPA), polycyclic aromatic hydrocarbons (PAHs), dioxins and dichlorodiphenyltrichloroethane (DDT), can affect the physiological function of adipose tissue by changing the volume or the number of adipocytes (11, 13, 14). In addition, alcohol and tetrachlorodibenzo-*p*-dioxin (TCDD) can be metabolized in adipose tissue and ultimately affect their toxicity (15, 16). The above studies demonstrate that adipose tissue not only is a simple repository of such lipophilic substances but also has complex biological functions. Complicated interactions may exist between lipophilic chemicals and adipose tissue.

As a lipophilic chemical, benzene is also reported to be involved in disrupting lipid metabolism, and adipose tissue is speculated to be involved in the regulation of benzene-induced hematotoxicity. It has been shown that the elimination rate of benzene decreased significantly in mice and people with high body fat content, and the body fat content of mice may affect the susceptibility to hematotoxicity caused by a high concentration of benzene (17). Our previous study also found that body fat content might be associated with the erythroid-related hematotoxicity caused by low concentration of benzene in petrochemical workers, and workers with lower or higher fat content were more sensitive to benzene-induced hematotoxicity (18). In addition, Sun et al. reported that benzene exposure can affect the oxidation of fatty acids which is one of the mechanisms of benzene-induced hematotoxicity (19, 20). All these findings suggest a probably complicated interaction between benzene and adipose tissue, which remains to be elucidated.

This study assessed the impact of benzene exposure on adipose tissue and adipocytes. Animal experiments were conducted using adult male C57BL/6J mice exposed to benzene at 0, 1, 10 and 100 mg/kg body weight (bw) by intragastric gavage for 4 weeks, and the effect of benzene on adipose tissues was examined. In addition, *in vitro* cell experiments using 3T3-L1 cells were also conducted to explore the effect of hydroquinone (HQ), a toxic metabolite of benzene, on adipocytes. The findings will shed light on the effect of benzene exposure on adipose tissue, and provide a powerful clue for exploring the mechanism underlying benzene-related metabolic disorders.

## Materials and Methods

### Animals and Materials

Adult male C57BL/6J mice (14 weeks old) were purchased from Liaoning Changsheng Biotechnology co., Ltd. (Benxi, China). Benzene was provided by Sigma-Aldrich. Hydroquinone was provided by Fluka. The blood routine test kit was provided by Drew Scientific (Miami Lakes, USA), and the kits for detecting triglyceride (TG), total cholesterol (TC), non-esterified fatty acid (NEFA), high-density lipoprotein cholesterol (HDL-C) and low-density lipoprotein cholesterol (LDL-C) were obtained from Nanjing Jiancheng Bioengineering Institute (Nanjing, China). Leptin and adiponectin ELISA kits were provided by Mlbio (Shanghai, China).

### Animal Experiment

The adult male C57BL/6J mice were housed in a specific pathogen-free (SPF) animal room in a 12 h dark/light cycle at 20 ± 3°C and 50% humidity. Mice were allowed free access to food and water. The mice were exposed to various doses of benzene and each group was housed separately in polypropylene cages with a maximum of five mice in each cage. Benzene exposure manner and doses were designed according to our previous study (21). Briefly, the mice were randomly divided into four groups (n = 11-12), and benzene was diluted in corn oil. Mice were administrated with benzene by oral gavage at doses of 0, 1, 10 and 100 mg/kg for a consecutive 4 weeks (6 times/week). The body weight and food intake of mice were monitored daily. This study was conducted in strict accordance with the guidelines for the care and use of laboratory animals issued by the State Science and Technology Commission of the People’s Republic of China. The experimental scheme was approved by the Laboratory Animal Ethics Committee, School of Public Health.

### Detection of Urinary S-Phenylmercapturic Acid 

Urinary SPMA detection was performed according to our previous protocol (21). Briefly, urine samples were collected using metabolic cages after the last gavage. Then after centrifugation at 3000 rpm for 5 min, the supernatant was collected for detecting the SPMA level by liquid chromatography/electrospray tandem mass spectrometry (LC-MS/MS) (Agilent, Santa Clara, USA). Urinary levels of SPMA were normalized to creatinine, which was measured by the creatinine detection kit (Nanjing Jiancheng Bioengineering Institute, Nanjing, China).

### Blood Routine Test and Biochemical Analysis

Blood was collected by inferior vena cava puncture. A blood routine test was conducted by an automatic blood cell analyzer according to our previous protocol (21). According to our previous study (21) and research conducted by Lan etal. (22). (23), cell counts of white blood cells (WBC), neutrophils, lymphocytes and monocytes showed high sensitivity to benzene-induced hematotoxicity. Therefore, in this study, these four indexes were tested preferentially to reflect the hematotoxicity of benzene. For biochemical analysis, plasma levels of TG, TC, HDL-C, LDL-C and NEFA were measured using commercial kits, respectively.

### Body Composition Analysis and Body Fat Distribution

The body composition of mice was measured by the Niumag small animal body composition analyzer (Suzhou Niumag Analytical Instrument Corporation, Suzhou, China), according to the manufacturer’s guidelines. Then mice were anesthetized with pentobarbital sodium, and diverse adipose depots were carefully dissected and weighed, including two white adipose tissues (WATs) and one brown adipose tissue (BAT). Epididymal and inguinal subcutaneous adipose depots were representative WATs, while interscapular brown fat was dissected as representative BAT. Then, we calculated the fat content of each adipose depot (fat content = fat pad weight/body weight × 100%) and WAT fat content (the sum of the above two WAT depots) for each mouse.

### Histological Analysis

The distal adipose tissue of the epididymis was fixed with 4% paraformaldehyde (PFA). Then tissues were sectioned after paraffin embedding and then stained with hematoxylin and eosin (H&E) (24). All tissue sections were observed under a biological microscope and photographed. The adipocytes of adipose tissue were analyzed using Image J software (National Institutes of Health, Bethesda, USA).

### *In Vitro* Experiment

*In vitro* experiments were conducted using adipocytes differentiated from preadipocyte 3T3-L1. The differentiation protocol was according to Hsu’s report with minor modifications (25). Briefly, two days post confluence the 3T3-L1 cells were incubated in adipogenesis-inducing medium (AIM) cocktails (DMEM medium containing 1 μmol/L dexamethasone, 0.5 mM IBMX, 10 μg/mL insulin, 1% penicillin, 1% streptomycin and 10% FBS) for 3 days. Next, cells were cultured in adipogenesis maintaining medium (AMM) (DMEM medium containing 10 μg/mL insulin, 1% penicillin, 1% streptomycin and 10% FBS) for 6 days with the medium changed every 3 days. Then cells were maintained in DMEM (1% penicillin, 1% streptomycin and 10% FBS) for another 2 days. Fully differentiation is usually achieved by day 11 (ID11). Then mature adipocytes were exposed to HQ at 0, 1, 5 and 25 μM for 24 hours, respectively. The doses of HQ treatment were designed according to our previous study and cytotoxicity was evaluated by trypan blue staining (26). The levels of intracellular lipid and released-TG were determined to reflect the lipid accumulation and lipolysis activity, respectively. The main genes related to lipid metabolism were also detected. The endocrine function of adipocytes was assessed by examining the levels of leptin and adiponectin at both mRNA and protein levels.

### Enzyme-Linked Immunosorbent Assay 

The plasma samples were centrifuged at 3000 rpm and 4°C for 20 min, then the supernatants were taken for measurement of blood lipids and adipokines according to the manuals of commercial kits. Cell culture medium was collected and centrifuged at 3000 rpm and 4°C for 20 min, then the supernatant was taken for measurement of adipokines according to the manuals of commercial kits.

### Real-Time Reverse Transcription Quantitative Polymerase Chain Reaction 

The mRNA levels of adipokines and lipometabolism-related genes were detected. Total RNA was isolated from epididymal fat tissues or adipocytes with TRIzol Reagent (Invitrogen, Carlsbad, USA). Then the Takara primescript™ RT reagent (Takara Biotechnology Co., Ltd., Dalian, China) was used to reverse transcribe 1 ng RNA. With Quantum Studio™ real-time PCR software, SYBR Green (TOYOBO Biotechnology Co., Ltd., Shanghai, China) was used for RT-qPCR. The target gene primer pairs were synthesized by Tsingke Biotechnology Co., Ltd. (Beijing, China). The primer sequence was shown in **Supplementary Table 1** (27–29). The 2^−ΔΔCt^ method was used to determine the relative gene expression (30).

### Statistical Analysis

Statistical analyses were performed in SPSS 25.0 software (IBM, Armonk, USA). All data were presented by mean ± standard deviation (SD). One-way analysis of variance (ANOVA) was used to compare the differences between groups. LSD multiple comparison test was used for pairwise comparison, and polynomial contrast procedure was conducted to analyze the changing trend. Pearson correlation coefficient was used to analyze the relationship between fat content or plasma adipokine levels, and peripheral blood leukocyte parameters. All tests were performed by a two-sided test, *p* < 0.05 was considered statistically significant.

## Results

### Benzene Exposure Induced Dyslipidemia

**Figure 1:**
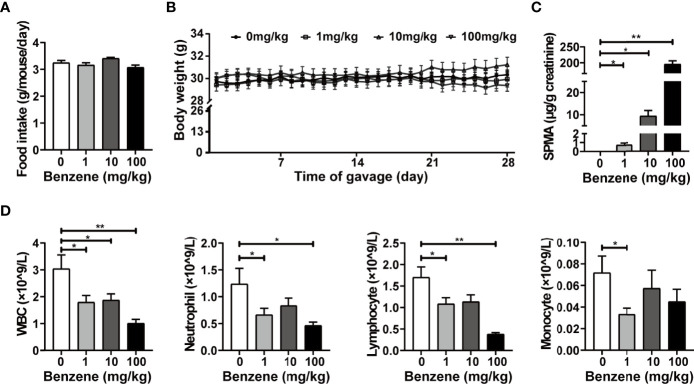
Exposure of benzene-induced hematotoxicity in mice. Average food intake **(A)** and body weight gain kinetic **(B)** during the period of benzene exposure were measured. The internal exposure level of benzene was reflected by urinary SPMA corrected by creatinine **(C)**. The count of peripheral blood cells **(D)** including white blood cells (WBC), neutrophils, lymphocytes and monocytes was determined after benzene exposure. Data are presented as mean ± SD (n = 11-12), and one way ANOVA were performed; *p < 0.05, ** *p* < 0.001 (compared to the control group).

Adult male C57BL/6J mice were exposed to benzene at doses of 0, 1, 10 and 100 mg/kg bw for four weeks. During the period of benzene treatment, no obvious changes in food intake and body weight were observed (**Figures 1A, B**). At the end of the benzene-treatment period, the urinary SPMA level of mice, corrected by creatinine, was increased in a dose-dependent manner (*p_trend_* < 0.001, **Figure 1C**). The urinary SPMA levels of 1 and 10 mg/kg groups were equivalent to that of adult male mice exposed to 1 and 10 ppm benzene for 6h/d, respectively (data not shown). Findings indicated that the cell count of WBC, neutrophils and lymphocytes decreased in a dose-dependent manner (all *p_trend_* < 0.05, **Figure 1D**). Exactly, compared with the control group, the WBC count of 1, 10 and 100 mg/kg group was decreased by 41.0% (*p* < 0.05), 38.4% (*p* < 0.05) and 66.7% (*p* < 0.001), respectively (**Figure 1D**). While the cell counts of neutrophils and lymphocytes showed significant decrease in 1 and 100 mg/kg (all *p* < 0.05, **Figure 1D**). These results were consistent with our previous study (21), indicating that we successfully established a benzene-induced hematotoxicity model in mice. Subsequently, the effect of benzene exposure on adipose tissue was observed in these mice.

In our study, the plasma levels of TG, TC, HDL-C, LDL-C and NEFA were also detected. Findings showed no significant changes in blood TG, HDL-C and LDL-C levels (**Table 1**). However, the plasma TC level decreased from 1 mg/kg dose group (*p*_trend_ < 0.05), and TC content in 100 mg/kg group was 14.3% lower than that of the control group (*p* < 0.05). More interestingly, the plasma NEFA level showed a significant upward trend from 1 mg/kg (*p*_trend_ < 0.05), with 20.6% higher in 100 mg/kg group than that of the control group (*p* < 0.05). As it is well-known that adipose tissue is the major source of NEFA, the increased blood NEFA level may suggest increased lipolysis upon benzene exposure. These results indicate that benzene exposure can induce dyslipidemia.

**Table 1:** Effect of benzene on biochemical indexes.

Biochemical indexes	0 mg/kg (n = 7)	1 mg/kg (n = 7)	10 mg/kg (n = 8)	100 mg/kg (n = 8)	*P*_trend_^a^
TG (mmol/L)	0.71 ± 0.20	0.65 ± 0.24	0.60 ± 0.19	0.58 ± 0.16	0.170
HDL-C (mmol/L)	2.82 ± 0.34	2.81 ± 0.46	2.92 ± 0.53	3.00 ± 0.40	0.368
LDL-C (mmol/L)	0.45 ± 0.19	0.27 ± 0.11	0.31 ± 0.18	0.42 ± 0.22	0.913
TC (mmol/L)	3.14 ± 0.26	2.90 ± 0.29	2.89 ± 0.46	2.69 ± 0.31*	0.023
NEFA (mmol/L)	0.73 ± 0.12	0.77 ± 0.08	0.77 ± 0.19	0.88 ± 0.07*	0.039

TG, triglyceride; HDL-C, high-density lipoprotein cholesterol; LDL-C, low-density lipoprotein cholesterol; TC, total cholesterol; NEFA, non-esterified fatty acid. Values represent mean ± SD; one way ANOVA was performed; ^a^ The test for trend was performed with a polynomial contrast procedure; * p < 0.05 (compared to the control group).

### Benzene Exposure Significantly Decreased Total Body Fat Content

**Figure 2:**
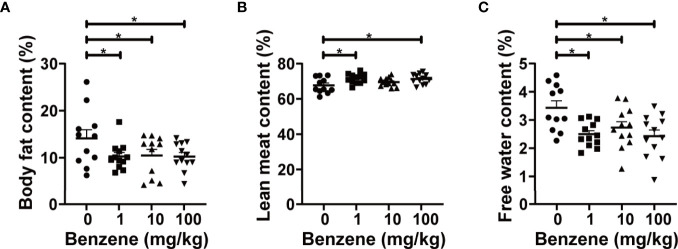
Effect of benzene on body composition. Body fat content **(A)**, lean meat content **(B)** and free water content **(C)** were measured to reflect body fat distribution after benzene treatment. Data are presented as mean ± SD (n = 11-12). One-way ANOVA were performed; *p < 0.05 (compared to the control group).

To explore the effect of benzene exposure on body fat content, a non-invasive body composition analysis was firstly conducted. The results showed obvious alterations in body composition after benzene exposure, with a significant decrease in body fat content. Compared with the control group, average body fat content in 1, 10 and 100 mg/kg groups decreased by 27.15%, 25.74% and 27.34% (all *p* < 0.05), respectively (**Figure 2A**). Meanwhile, higher content of lean meat in 1 and 100 mg/kg groups (both *p* < 0.05, **Figure 2B**) and lower content of free water in all three benzene exposed groups compared with the control group (all *p* < 0.05, **Figure 2C**) were observed. These results indicate that benzene exposure can alter the body composition of mice and dramatically decrease body fat content even in the 1 mg/kg group.

### The Reduced Body Fat Content Was Mainly Due to a Significant Decrease in WAT Fat Content

**Figure 3:**
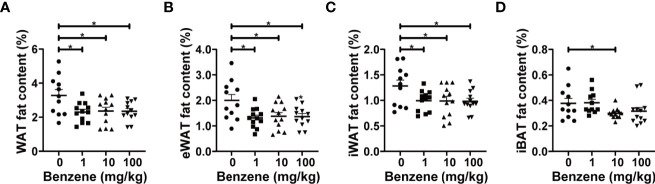
Effect of benzene on fat content. The body fat distribution was reflected by the fat content of representative adipose depots **(A–D)** including epididymal white adipose tissue (eWAT), inguinal white adipose tissue (iWAT), white adipose tissue (WAT) and interscapular brown adipose tissue (iBAT). All parameters were measured after four-week exposure. Data are presented as mean ± SD (n = 11-12). One-way ANOVA were performed; *p < 0.05 (compared to the control group).

Based on the significant decrease in total body fat content upon benzene exposure, we further investigated which adipose tissue subtype is more sensitive to benzene toxicity. The following results indicated that WAT may be more vulnerable than BAT. Significant decreases in various subtypes of WAT fat content were observed even at the lowest dose group, 1 mg/kg (**Figures 3A–C**), while the content of interscapular brown adipose tissue (iBAT) only decreased in the 10 mg/kg group (20.9% lower than the control group, *p* < 0.05, **Figure 3D**). Compared with the control group, the eWAT fat content in 1 mg/kg, 10 mg/kg and 100 mg/kg groups decreased by 34.4%, 31.1% and 31.7% (**Figure 3B**), and the inguinal white adipose tissue (iWAT) fat content in 1 mg/kg, 10 mg/kg and 100 mg/kg groups decreased by 22.6%, 22.9% and 23.4%, respectively (all *p* < 0.05, **Figure 3C**). Total WAT fat content was calculated by adding the content of the above two white adipose depots, which was 29.8%, 27.9% and 28.4% lower than the control group in 1 mg/kg, 10 mg/kg and 100 mg/kg groups (all *p* < 0.05, **Figure 3A**), respectively. These findings indicate that the white subtype of adipose tissue is more sensitive to benzene-induced toxicity, even at the lowest concentration of 1 mg/kg, which may account for the loss of body fat after benzene exposure.

### Benzene Exposure Could Reduce the Cell Size of Adipocytes in WAT

**Figure 4:**
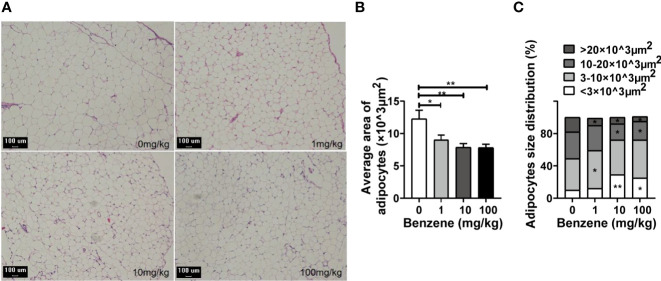
Effect of benzene on epididymal white adipose tissue (eWAT). **(A)** Representative pictures of the eWAT section with HE staining (100×, bar = 100 μm). The average area of adipocytes of each group **(B)** and adipocytes size distribution **(C)** were analyzed. Data are presented as mean ± SD (n = 7-8). One way ANOVA were performed; *p < 0.05, ** *p* < 0.001 (compared to the control group).

Given the adverse effect of benzene exposure on WAT, we next analyzed the pathological changes of WAT. Histological analysis was conducted using the eWAT, and shrinking adipocytes were observed (**Figures 4A, B**). As shown in **Figure 4B**, the area of adipocytes in 1 mg/kg, 10 mg/kg and 100 mg/kg group was 26.5% (*p* < 0.05), 35.8% (*p* < 0.001) and 36.2% (*p* < 0.001) less than that of the control group, respectively. For further information, we analyzed the adipocyte composition of eWAT. Findings indicated that despite benzene exposure having no obvious effect on the total number of adipocytes, the percentage of small adipocytes increased in a dose-dependent manner (*p*_trend_ < 0.001) while that of large adipocytes decreased from the 1 mg/kg group (**Figure 4C**). Compared with the control group, the percentage of small adipocytes with an area of (3-10) ×10^3^ μm^2^ in the 1 mg/kg group was 29.9% higher than that of the control group (*p* < 0.05, **Figure 4C**). Meanwhile, the percentage of the large adipocytes with an area > 20×10^3^ μm^2^ in the 1 mg/kg group decreased by 63.4% when compared to the control group (*p* < 0.05, **Figure 4C**). Similarly, increasing in large adipocyte percentage and decreasing in small adipocyte percentage, were observed in 10 mg/kg and 100 mg/kg groups, respectively (both *p* < 0.05, **Figure 4C**). In summary, these results exhibited that although benzene exposure has no impact on the total number of adipocytes in eWAT, a considerable number of adipocytes became smaller upon benzene exposure. All these findings suggest that an imbalance of lipid homeostasis may occur after benzene exposure, and the shrinking adipocytes may explain why benzene exposure decreased the WAT fat content in mice even at the lowest dose.

### Benzene Exposure Altered Critical Lipometabolism Genes in WAT

**Figure 5:**
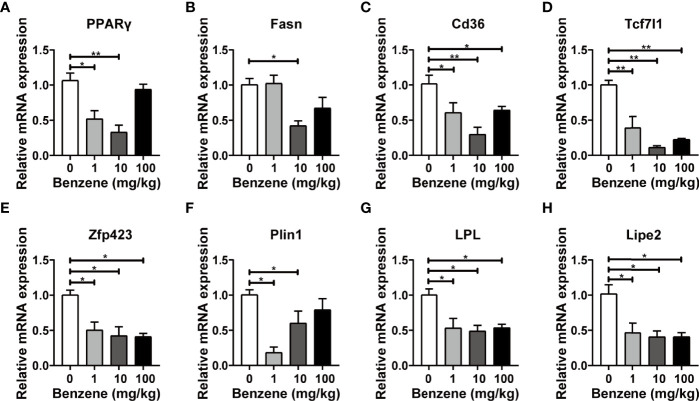
Effect of benzene on mRNA levels of lipid metabolism genes in epididymal white adipose tissue (eWAT). Adult male C57BL/6J mice were exposed to benzene at doses of 0, 1, 10 and 100 mg/kg for 4 weeks. The mRNA expression of genes involved in lipogenesis **(A–E)** and lipolysis **(F–H)** were examined in eWAT after benzene exposure. Data are presented as mean ± SD (n = 3-6) and one way ANOVA were performed; *p < 0.05, **p < 0.001 (compared to the control group).

Based on the above findings, we further investigated the expression levels of critical genes involved in pivotal lipid metabolism signaling. Peroxisome proliferator-activated nuclear receptor gamma (*PPARγ*) is abundantly expressed in adipose tissue and plays a key role in the regulation of adipogenesis and lipometabolism, especially in lipid homeostasis regulation (31, 32). In this study, *PPARγ* mRNA expression was significantly down-regulated in 1 mg/kg (*p* < 0.05) and 10 mg/kg groups (*p* < 0.001, **Figure 5A**). Another three critical genes involved in lipogenesis, *Cd36*, *Tcf71* and *Zfp43*, *were* all down-regulated in 1 mg/kg,10 mg/kg and 100 mg/kg groups (all *p* < 0.05, **Figures 5C–E**). On the other hand, three pivotal genes involved in lipolysis were also detected. Significant down-regulation of *LPL* and *Lipe2* mRNA expression was observed in 1 mg/kg, 10 mg/kg and 100 mg/kg groups (all *p* < 0.05, **Figures 5G, H**). These results showed that both lipid synthesis and lipid lysis signalings were attacked by benzene exposure, and the disturbance of lipid metabolism signalings may explain why the adipocytes become smaller in benzene exposed groups.

### HQ Exposure Impacted Lipid Homeostasis in 3T3-L1 Adipocytes

**Figure 6:**
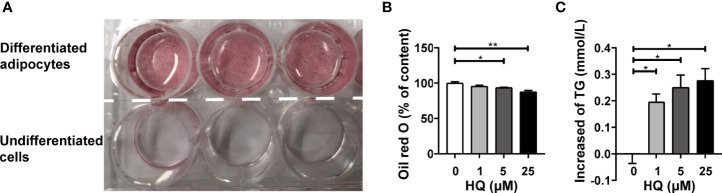
Effect of HQ on 3T3-L1 adipocytes. **(A)** The adipocytes were successfully differentiated at ID11 and stained with oil red O. The upper three dishes showed mature adipocytes differentiated from preadipocytes 3T3-L1, and the lower three dishes showed undifferentiated cells. The fat content of adipocytes **(B)** and adipocytes-released TG **(C)** was determined after 24-hour exposure to HQ. Data are presented as mean ± SD (n = 3-6). One way ANOVA were performed; *p < 0.05, ** *p* < 0.001 (compared to the control group).

In our study, mature adipocytes were obtained from 3T3-L1 cells. The findings indicated that up to ID11 more than 95% of cells were fully differentiated into adipocytes (**Figure 6A**). Trypan blue staining showed that 24h-HQ-exposure had no obvious cytotoxicity (data not shown) on adipocytes even at 25μM. However, the lipid content decreased significantly in HQ-treated cells (**Figure 6B**), and the level of TG released from cells into the medium increased in a dose-dependent manner (*p_trend_* < 0.05, **Figure 6C**). These results indicated that HQ exposure might increase lipolysis and subsequently reduce the lipid content of adipocytes, which was consistent with our *in vivo* findings.

**Figure 7:**
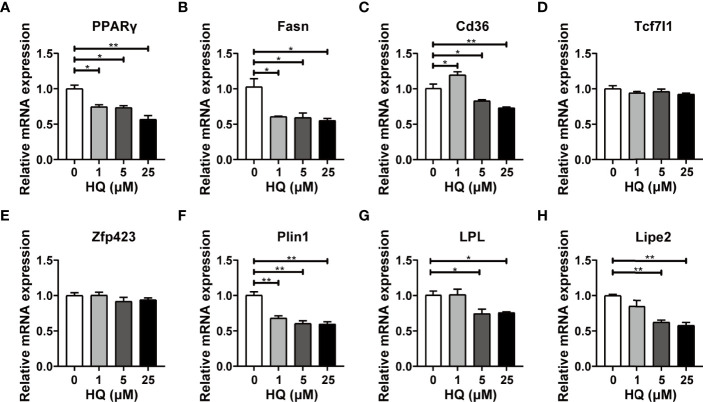
Effect of HQ on mRNA levels of lipid metabolism genes in adipocytes. The mRNA expression of genes involved in lipogenesis **(A–E)** and lipolysis **(F–H)** were examined in 3T3-L1 adipocytes after 24-hour exposure to HQ, respectively. Data are presented as mean ± SD (n = 3-6). One way ANOVA were performed; *p < 0.05, ** *p* < 0.001 (compared to the control group).

The RT-qPCR results displayed similar changes in *PPARγ* mRNA expression to that of *in vivo* experiment. Exactly, *PPARγ* mRNA level was significantly down-regulated in 1 μM (*p* < 0.05), 5 μM (*p* < 0.05) and 25 μM groups (*p* < 0.001, **Figure 7A**). Four crucial genes related to lipogenesis were also detected, among which *Fasn* was down-regulated in all HQ-exposed groups (all *p* < 0.05, **Figure 7B**). However, *Cd36* mRNA expression was up-regulated in 1 μM group (*p* < 0.05) and down-regulated in 5 μM (*p* < 0.05) and 25 μM groups (*p* < 0.001, **Figure 7C**). In addition, no significant change was observed in *Tcf7l1* and *Zfp423* (**Figures 7D, E**). Three pivotal genes involved in lipolysis were also detected. Significant down-regulation of *Plin1* mRNA expression was observed in all benzene-exposed groups (all *p* < 0.001, **Figure 7F**), while the mRNA expression of *LPL* (both *p* < 0.05, **Figure 7G**) and *Lipe2* (both *p* < 0.001, **Figure 7H**) was significantly down-regulated in 5 μM and 25 μM groups. The results showed that HQ exposure could disturb the expression of critical lipometabolism genes. All these findings suggest that the intermediate metabolite of benzene disturbs the lipid homeostasis of adipocytes, which is in accordant with the findings of *in vivo* experiment.

### Benzene Exposure Impacted the Endocrine Function of WAT

**Figure 8:**
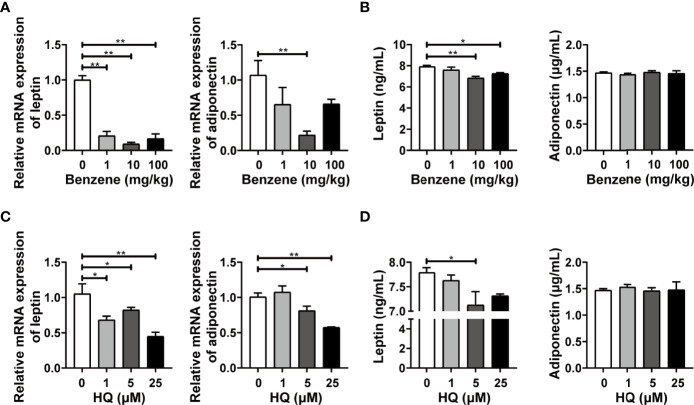
The endocrine activity of white adipose tissue (WAT) and *in vitro* adipocytes. Adult male C57BL/6J mice were exposed to benzene at doses of 0, 1, 10 and 100 mg/kg for 4 weeks. Then the mRNA levels of leptin and adiponectin were detected in eWAT **(A)**, and those of plasma levels were detected using ELISA **(B)** (n = 5-6). Mature adipocytes were exposed to HQ at 0, 1, 5 and 25 μM for 24 hours. Then the mRNA levels of leptin and adiponectin were detected **(C)**, and the medium content of leptin and adiponectin was examined using ELISA **(D)** (n = 3-4). Data are presented as mean ± SD. One way ANOVA were performed; *p < 0.05, ** *p* < 0.001 (compared to the control group).

The above findings indicated that benzene exposure can induce obvious adverse effects on WAT. Since WAT has important roles in regulating the function of distant organs or related diseases in an endocrine manner, we further evaluated the effect of benzene exposure on the endocrine function of WAT. The biomarkers for the endocrine activity of adipose tissues, leptin and adiponectin, were detected. Results showed that the mRNA expression level of adiponectin decreased significantly in 10 mg/kg group compared with the control group (*p* < 0.001, **Figure 8A**), while no significant change was observed in plasma adiponectin level (**Figure 8B**). By contrast, leptin exhibited higher sensitivity. Compared with the control group, leptin mRNA expression in 1 mg/kg, 10 mg/kg and 100 mg/kg group decreased by 79.20% (*p* < 0.05), 90.83% (*p* < 0.001) and 42.00% (**Figure 8A**), respectively. In addition, when compared to the control group, the plasma leptin level in 10 mg/kg and 100 mg/kg (both *p* < 0.05) groups also showed significant decrease (**Figure 8B**). Similar findings were obtained from *in vitro* experiments. Compared with adiponectin, the biomarker leptin showed better sensitivity. The leptin mRNA expression was down-regulated in adipocytes exposed to HQ at 1 μM (*p* < 0.05), 5 μM (*p* < 0.05) and 25 μM groups (*p* < 0.001, **Figure 8C**), while significant decrease in culture medium content of leptin was observed from 5 μM group (*p* < 0.05, **Figure 8D**). Together with the *in vivo* findings, these results indicate that exposure to benzene or its toxic metabolite HQ disturbs the endocrine function of white adipocytes.

### Correlation of Peripheral Blood Parameters With WAT Contents and Adipokine Levels

As WAT is reported to be involved in hematological diseases through endocrine, we next analyzed the relationship between fat content (body fat content, iBAT fat content, eWAT fat content, iWAT fat content and WAT fat content) and plasma adipokines (leptin and adiponectin) levels with peripheral blood leukocyte parameters (WBC, neutrophil, lymphocyte and monocyte), respectively. As shown in **Table 2**, there were significant positive relationships between total body fat content and WBC, as well as neutrophil and monocyte count (all *p* < 0.05). And the WAT fat content, especially eWAT fat content, also showed positive correlations with WBC, neutrophil and monocyte count (*p* < 0.05). In addition, significant positive correlations between plasma leptin levels and WBC counts, as well as lymphocyte counts were also observed (both *p* < 0.05) (**Table 3**). The findings suggest that WAT, especially the visceral WAT, may affect the blood routine during benzene exposure, and leptin may play an important role in this process.

**Table 2:** Pearson correlation analysis between fat content and blood leukocyte parameters.

	Body fat content (%)	iBAT fat content (%)	eWAT fat content (%)	iWAT fat content (%)	WAT fat content (%)	WBC (×10^9^/L)	Neutrophil(×10^9^/L)	Lymphocyte(×10^9^/L)	Monocyte(×10^9^/L)
Body fat content (%)	1	-0.218	0.912^**^	0.882^**^	0.928^**^	0.343^*^	0.329^*^	0.246	0.307^*^
iBAT fat content (%)		1	-0.114	-0.083	-0.107	-0.223	-0.242	-0.166	-0.219
eWAT fat content (%)			1	0.873^**^	0.985^**^	0.347^*^	0.330^*^	0.263	0.360^*^
iWAT fat content (%)				1	0.944^**^	0.265	0.210	0.239	0.147
WAT fat content (%)					1	0.330^*^	0.299^*^	0.264	0.296^*^
WBC (×10^9^/L)						1	0.857^**^	0.927^**^	0.639^**^
Neutrophil (×10^9^/L)							1	0.678^**^	0.683^**^
Lymphocyte (×10^9^/L)								1	0.498^**^
Monocyte (×10^9^/L)									1

iBAT, interscapular brown adipose tissue; eWAT, epididymal white adipose tissue; iWAT, inguinal white adipose tissue; WAT, white adipose tissue; WBC, white blood cell. ^*^ Statistically significant correlation at p < 0.05; ^**^ Statistically significant correlation at p < 0.001.

**Table 3:** Pearson correlation analysis between plasma adipokine level and blood leukocyte parameters.

	WBC (×10^9^/L)	Neutrophil (×10^9^/L)	Lymphocyte (×10^9^/L)	Monocyte (×10^9^/L)	Leptin (mmol/L)	Adiponectin (mmol/L)
WBC (×10^9^/L)	1	0.857^**^	0.927^**^	0.639^**^	0.344^*^	0.101
Neutrophil (×10^9^/L)		1	0.678^**^	0.683^**^	0.206	0.164
Lymphocyte (×10^9^/L)			1	0.498^**^	0.348^*^	0.047
Monocyte (×10^9^/L)				1	0.114	0.132
Leptin(mmol/L)					1	-0.010
Adiponectin(mmol/L)						1

WBC, white blood cell. ^*^ Statistically significant correlation at p < 0.05; ^**^ Statistically significant correlation at p < 0.001.

## Discussion

Benzene is a ubiquitous lipophilic pollutant with multi-organ toxicity. Adipose tissue is routinely considered to be one of the main organs in which benzene accumulates. Interestingly, our study shows that benzene exposure can not only cause hematotoxicity but also impact the physiology of adipose tissue, manifesting as lipodystrophy of WAT and altered endocrine activity. In addition, significant correlations between WAT fat content, plasma leptin and hematotoxicity were also observed. This study shed light on the novel aspect that benzene exposure could disturb lipid homeostasis and endocrine function of WAT, which might be associated with benzene-induced hematotoxicity. The findings provide a new clue for further studying benzene toxicity from the perspective of adipose tissue.

Benzene is long known as a ubiquitous volatile organic pollutant associated with hematotoxicity. Given serious health hazards, many countries have defined benzene exposure limits. The latest national standard in China established the permissible concentration-time weighted average (PC-TWA) of 6 mg/m^3^ (1.85 ppm) (33), while the occupational standard in America is 1 ppm (3.25 mg/m^3^) (34). Formerly, the focus of studies was on hematotoxicity and leukemia induced by benzene exposure at relatively high levels. However, many studies reported that hematotoxicity may happen even if the benzene exposure level was below 1 ppm (22, 35). Thus nowadays, concerns have shifted to damages induced by benzene exposure at relatively much lower levels. In addition, since benzene also exists in water and processed food (36, 37), oral administration of benzene is necessary for experimental design. In this study, the lowest benzene exposure dose was 1 mg/kg bw by intragastric administration, with the urinary SPMA level equivalent to that of mice exposed to 1 ppm benzene for 6h/d. The results indicated that mice exposed to benzene at 1 mg/kg showed significant hematotoxicity, demonstrating as significantly decreased cell counts of WBC, neutrophil, lymphocyte and monocyte. Interestingly, apart from the routine hematotoxicity, we observed a significant decrease in total body fat content, especially the WAT fat content, from the 1 mg/kg group. Meanwhile, the NEFA level showed a significant upward trend in benzene-exposed groups, from 1 mg/kg (*p*_trend_ < 0.05), which may be related to the release of NEFA from adipose tissue decomposition. The following pathological analysis indicated that the percentage of small adipocytes significantly increased from the 1 mg/kg group, while that of large adipocytes significantly decreased. These findings suggest that benzene causes lipodystrophy in normal-weight mice. Unlike fat loss in obese mice, lipodystrophy in normal mice indicates lipometabolism disorder, which is harmful to health.

Benzene exposure is reported to be involved in lipid metabolism. Sun et al., previously conducted a plasma metabolomics investigation in Chinese benzene-exposed workers with low white blood cell count and identified nine differential metabolites, including the lipid metabolism pathway (20). Besides, their research using male C3H/He mice indicated that benzene exposure might induce fatty acid oxidation which is involved in hematotoxicity (38). Recently, this team investigated the effects of benzene on gut microbiota and metabolism in mice exposed to 0, 6, 30 and 150 mg/kg benzene by subcutaneous injection for 30 days. And findings showed that several metabolic pathways were significantly influenced by benzene exposure, suggesting that benzene exposure caused dysbiosis of the gut microbiota and metabolic disorders in mice, including steroid biosynthesis (39). All these findings suggest that benzene exposure could disturb metabolic homeostasis, especially fat acid metabolism. In this study, we also found the disorder of critical lipometabolism genes involved in both lipogenesis and lipolysis *in vivo* and *in vitro*. These results suggest that benzene disturbs lipid homeostasis *via* dysregulating the vital molecules involved in lipid metabolism, ultimately resulting in WAT dystrophy.

It is reported that adipose tissue defects, including severe obesity and lipodystrophy, can lead to insulin resistance (IR) and related metabolic disorders (7). More recently, a growing body of evidence indicates that many environmental chemicals can interfere with adipose tissues, impacting body weight, lipid profile, and related signaling pathways (40–42). And such disturbance of adipose tissue finally results in physiological disorders and diseases such as obesity, cardiovascular diseases, and type 2 diabetes (T2D) (40–42). Some endocrine disrupting chemicals (EDCs), e.g. bisphenol A (BPA) (43) and tributyltin (TBT) (44), are reported to have such properties. Besides, common pollutants such as arsenic (45) and PM_2.5_ (46) are also reported to be related to higher body weight and T2D. Studies have indicated that some of these chemicals influence lipid metabolism *via* activating *PPARγ* and subsequently increase the number of adipocytes and/or the amount of fat stored in adipocytes by altering energy metabolism pathways and/or food intake (41, 47). Interestingly, a contradictory effect on adipose tissue was also reported. For example, TCDD, a highly lipophilic pollutant, is reported to accumulate in WAT and cause lipodystrophy (15). A weight loss of female zebrafish was also observed after DDT exposure (48). Similar results were obtained in our study that benzene exposure could also induce lipodystrophy of WAT, accompanied by increasing trend of circulating NEFA level. Although obesity has been considered harmful to human health for a long time, it doesn’t mean that the lower the body fat, the better. It has been reported that lipodystrophy predisposes the patients to IR and related complications such as diabetes and hepatic steatosis (49, 50). What’s more, plasma NEFA is also reported to play an important role in the induction of IR (51). Interestingly, many studies have reported that benzene exposure may cause IR (3, 4, 52). Given the previous studies and our findings, we speculate that benzene exposure might impact the physiology of white adipose tissue, which in turn induces the related metabolism disorders at the system level. However, more studies are necessary to elucidate the underlying mechanism.

Recent studies reported that WAT could release hormones and adipokines to regulate appetite, metabolism and other organ functions (53–55). Some adipokines are reported to be related to hematopoiesis, such as leptin and adiponectin (56, 57). Leptin and adiponectin are two important adipokines usually used as biomarkers for evaluating the endocrine function of adipocytes (58). It is reported that leptin and adiponectin can exert an antiapoptotic effect on neutrophils (59, 60). In addition, leptin can also stimulate leukocyte production (61). All these studies suggested that leptins may be involved in hematopoiesis. In this study, significant decreases in leptin at both mRNA and protein levels were observed *in vitro* and *in vivo.* In addition, significant positive correlations between plasma leptin level and cell counts of WBC and lymphocyte were observed, respectively. Our findings are in accordance with these previous studies. In summary, our findings suggest that leptin might be involved in benzene-induced hemopoietic toxicity, and the results provide a new clue for further studying the effect of adipose tissue on hematotoxicity during benzene exposure.

This study is of great significance for a comprehensive understanding of benzene toxicity and better health risk assessment in the future. However, there is a limitation that although we have observed adverse effects of benzene on adipose tissue, the underlying mechanism remains to be further studied. Besides, although we observed a significant correlation between WAT content, plasma leptin level, and distinct cell counts, the exact mechanism remains unclear. Thus, in the future, detailed studies should be conducted to elucidate the above issues.

## Conclusion

In this work, the effect of benzene exposure on adipose tissue was preliminarily investigated. The findings indicated that adipose tissue is not just a reservoir of benzene. Benzene exposure could also induce lipodystrophy and alter endocrine activity of WAT, which in turn may affect the hematotoxicity of benzene *via* the adipokine manner. In conclusion, this research is of great significance for further studying the toxicity of benzene and the relationship between environmental pollutants and metabolic diseases.

## Data Availability Statement

The raw data supporting the conclusions of this article will be made available by the authors, without undue reservation.

## Ethics Statement

The animal study was reviewed and approved by the Laboratory Animal Ethics Committee, School of Public Health, Sun Yat-sen University.

## Author Contributions

XX designed the study. YC, ZM and PJ conducted the laboratory work. YC drafted the manuscript. YC, ZM, JZ, ZL, QL and ZH contributed to data analysis and statistics. DL, LQ, LC, QW, GD and YX contributed to resources. ZH, DL, WG, WC, XX and YX added expert inputs to the original manuscript. All authors further reviewed, edited, and approved the final manuscript. All authors contributed to the article and approved the submitted version.

## Funding

This work was funded by the National Natural Science Foundation of China (81973076; 81973006; 81602877).

## Conflict of Interest

The authors declare that the research was conducted in the absence of any commercial or financial relationships that could be construed as a potential conflict of interest.

## Publisher’s Note

All claims expressed in this article are solely those of the authors and do not necessarily represent those of their affiliated organizations, or those of the publisher, the editors and the reviewers. Any product that may be evaluated in this article, or claim that may be made by its manufacturer, is not guaranteed or endorsed by the publisher.
