# Supplementary material for: Benzene Exposure Leads to Lipodystrophy and Alters Endocrine Activity In Vivo and In Vitro
Source: Front Endocrinol (Lausanne). 2022 Jul 13;13:937281. doi: 10.3389/fendo.2022.937281 (PMC9326257; doi:10.3389/fendo.2022.937281)
Supplement: Supplementary file 1 [file Table_1.docx]

Supplementary Material

**Supplementary Table 1** Primer sequence for RT-qPCR

| Gene | Forward (5’-3’) | Reverse (3’-5’) |
| --- | --- | --- |
| *GAPDH* | GGCAAATTCAACGGCACA | GTTAGTGGGGTCTCGCTCCTG |
| *PPARγ* | GAAAGACAACGGACAAATCACC | GGGGGTGATATGTTTGAACTTG |
| *Fasn* | CCCTGACCAAGGTGCTGTTA | GGATCTCAGGGTTGGGGTTG |
| *Cd36* | CAGAGTTCGTTATCTAGCCAAGGAA | CATTGGGCTGTACAAAAGACACA |
| *Tcf7l1* | CCAGCACACTTGTCCAACAAA | AGCGGGTGCATGTGATGA |
| *Zfp423* | CCGCTGTGTGGTCTGTATGC | ATGTGAAAGGTGCCATGGATCT |
| *Plin1* | AGACTGAGGTGGCGGTCT | TTCTCCTGCTCAGGGAGGTCT |
| *LpL* | CATGGATGGACGGTAACGGG | TTCTCTCTTGTACAGGGCGG |
| *Lipe2* | AGGGAGGGCCTCAGCG | GTCTTCTGCGAGTGTCACCA |
| *PPARα* | GGGAACTTAGAGGAGAGCCAAG | CCATGTTGGATGGATGTGGC |
| *SCD1* | TTCTTGCGATACACTCTGGTGC | CGGGATTGAATGTTCTTGTCGT |
| *FATP2* | CGAGACGAGACGCTCACCTA | ACGAATGTTGTAGTTGAGGCAC |
| *Leptin* | TGTTCAAGCAGTGCCTATCCA | GAAGCCCAGGAATGAAGTCCA |
| *Adiponectin* | CAGTGGATCTGACGACACCA | AACGTCATCTTCGGCATGACT |
